# Hemocyte phagosomal proteome is dynamically shaped by cytoskeleton remodeling and interorganellar communication with endoplasmic reticulum during phagocytosis in a marine invertebrate, *Crassostrea gigas*

**DOI:** 10.1038/s41598-020-63676-3

**Published:** 2020-04-20

**Authors:** Fan Mao, Huawei Mu, Nai-Kei Wong, Kunna Liu, Jingchen Song, Jianwen Qiu, Yue Lin, Xiangyu Zhang, Duo Xu, Zhiming Xiang, Jun Li, Yang Zhang, Ziniu Yu

**Affiliations:** 1 CAS Key Laboratory of Tropical Marine Bio-resources and Ecology and Guangdong Provincial Key Laboratory of Applied Marine Biology, South China Sea Institute of Oceanology, Chinese Academy of Science, Guangzhou, China; 2 Innovation Academy of South China Sea Ecology and Environmental Engineering, Chinese Academy of Sciences, ISEE, CAS, Guangzhou, China; 3 Southern Marine Science and Engineering Guangdong Laboratory (Guangzhou), Guangzhou, China; 4 School of Life Sciences, University of Science and Technology of China, Hefei, China; 5 Department of Infectious Diseases, Shenzhen Third People’s Hospital, The Second Hospital Affiliated to Southern University of Science and Technology, Shenzhen, China; 6 College of Oceanology, South China Agricultural University, Guangzhou, China; 7 Croucher Institute for Environmental Sciences and the Department of Biology, Hong Kong Baptist University, Hong Kong, China

**Keywords:** Immunology, Molecular biology

## Abstract

Phagosomes are task-force organelles of innate immune systems, and evolutionary diversity and continuity abound in the protein machinery executing this coordinately regulated process. In order to clarify molecular mechanisms underlying phagocytosis, we studied phagocyte response to beads and *Vibrio* species, using hemocytes of the Pacific oysters (*Crassostrea gigas*) as a marine invertebrate model. Phagosomes from different stages of phagocytosis were isolated by density-gradient centrifugation, and more than 400 phagosome-associated proteins were subsequently identified via high-throughput quantitative proteomics. In modeling key networks of phagosomal proteins, our results support the essential roles of several processes driving phagosome formation and maturation, including cytoskeleton remodeling and signal transduction by Rab proteins. Several endoplasmic reticulum (ER)-associated proteins were identified, while live cell imaging confirms an apparent intimate interaction between the ER and phagosomes. In further quantitative proteomic analysis, the signal transducers *Cg*RhoGDI and *Cg*PI4K were implicated. Through experimental validation, *Cg*RhoGDI was shown to negatively regulate actin cytoskeleton remodeling in the formation of oyster phagosomes, while *Cg*PI4K signaling drives phagosome maturation and bacterial killing. Our current work illustrates the diversity and dynamic interplay of phagosomal proteins, providing a framework for better understanding host-microbe interactions during phagosome activities in under-examined invertebrate species.

## Introduction

The highly regulated cellular process of engulfing extracellular foreign particles is termed phagocytosis. It is characterized by complex cascades of events driving actin cytoskeleton rearrangement, vesicle formation, and internalization large particles into specialized compartments, culminating in the coordinated execution of physiological functions, such as host defense, immune response, macromolecular transport, metabolic adaptation, signal transduction, etc.^1–4^. In eukaryotes, phagocytic internalization of large particles is set in motion by particle binding to cell surface receptors, which cues the cell for reorganizing its plasma membrane and skeletal elements, ultimately leading to endocytosis of foreign particles and phagosome formation. Phagocytic phagosomes are a professionally dedicated organelle sequentially regulated by the formation of a phagocytic cup and then maturation from early to late phagosomes via fusion with vesicles of different types of endosomes^5–9^. Being an integral part of phagocytosis, phagosome formation is prerequisite for the killing and degrading of intracellular pathogens. Typically, mature phagosomes possess an internal pH between 4.5 and 5 and form a chemical niche for a series of recruited hydrolases^10,11^. Pathogens such as bacteria are essentially not killed by the act of internalization itself, but by an immunologically hostile luminal environment of mature phagosomes^6,12^. A number of factors have been shown to critically contribute to the phagosome maturation process, including phosphoinositides and other lipids, small GTPases, etc.^13^.

An interplay between extrinsic cues and intrinsic response of the phagocytic phagosome determines the complexity and dynamic variability of its protein composition. Our current understanding on immunity has depended much on evidence from terrestrial organisms such as human and Drosophila, whereas in marine invertebrates, immunity is achieved almost exclusively by innate immune processes whose governing principles are at once evolutionarily ancient and obscure. Notably, hemocytes in marine invertebrates constitute a principal effector cell population ideal for studying phagocytic dynamics. They also critically determine the health and disease of ecologically valuable species such as oysters, being a frequent target of pathogenic Vibrio species including *V. parahemolyticus*. In order to provide a theoretical framework for understanding phagocytic dynamics at work, it is imperative to employ large-scale, high-throughput, quantitative proteomic analyses to make available new data at the systems level for studying phagosomes. To this date, phagosome proteomes in human and mouse have been extensively explored^14–16^, whose focus includes differences between phagocytic cell types and cell response to cytokines (interferon-γ). Time- and space-resolved proteomic analysis of isolated phagosomes across species could be very useful for characterizing the functional machinery underpinning phagocytosis, including cell surface proteins, membrane transporters, endoplasmic reticulum proteins, mitochondrial proteins, hydrolases, GTPases, cytoskeleton and signal transducers^14^. Hydrolases, typically of lysosomal origin, are continuously transferred into the phagosomes, suggesting that the phagosomal and lysosomal systems engage each other in multiple interactions^17,18^.

Previously, proteomic evidence has described how primitive heterotrophic eukaryotes like *Tetrahymena thermophile* deploy phagocytosis as a means of nutrient ingestion in non-immunological contexts^19^. In most coelomate invertebrates including mollusks, annelids, arthropods, echinoderms, and tunicates, a sophisticated circulatory system has evolved, rich in hemocytes^20^. Nevertheless, mechanistic information concerning how invertebrate phagosomal proteins carry out their function in phagocytosis remains very limited. The Pacific oyster (*Crassostrea gigas*), a filter-feeding invertebrate, is exposed constantly to extreme environments that contain multitudes of commensals and pathogens^21,22^. *Vibrio* is widely found in seawater and estuary environmental waters, which is one of the main pathogens in marine aquaculture and can cause diseases in a variety of cultured animals such as fish, shrimp and shellfish^23^. *Vibrio* is also an important pathogen of humans, which can cause gastroenteritis, sepsis, cellulitis leading to necrotizing soft tissue infection in humans^24^. To compensate for a lack of adaptive immune components, oyster have evolved a relatively efficient mechanism for containing microbes and sustaining organism health, based on cellular and humoral immune responses^22,25^. Cellular immunity mainly relies on circulating hemocytes as primary immune effector cells, which display extraordinary phagocytic plasticity and versatility mirroring that of mammalian macrophages and neutrophils. A wide range of microorganisms and inorganic particles can be phagocytized by oyster hemocytes, including *vibrio*. Thus far, physiological and behavior details abound for the roles of phagocytosis against microbial infection in oysters. Yet at the systems level, a comprehensive understanding is still wanting, concerning the molecular determinants and dynamics of phagosome formation and maturation in marine invertebrates such as oysters. In this present study, we engaged in large-scale quantitative proteomics analysis with early and late phagosomes from oyster hemocytes, as an attempt to provide new insights into these evolutionarily vital processes, as well as affording a better understanding of how marine invertebrates utilize their first-line immune weaponry to ward off enteropathogens (such as *Vibrio spp*.) of interest to human diseases.

## Results

### Determination of phagosomal maturation in C. gigas hemocytes

**Figure 1:**
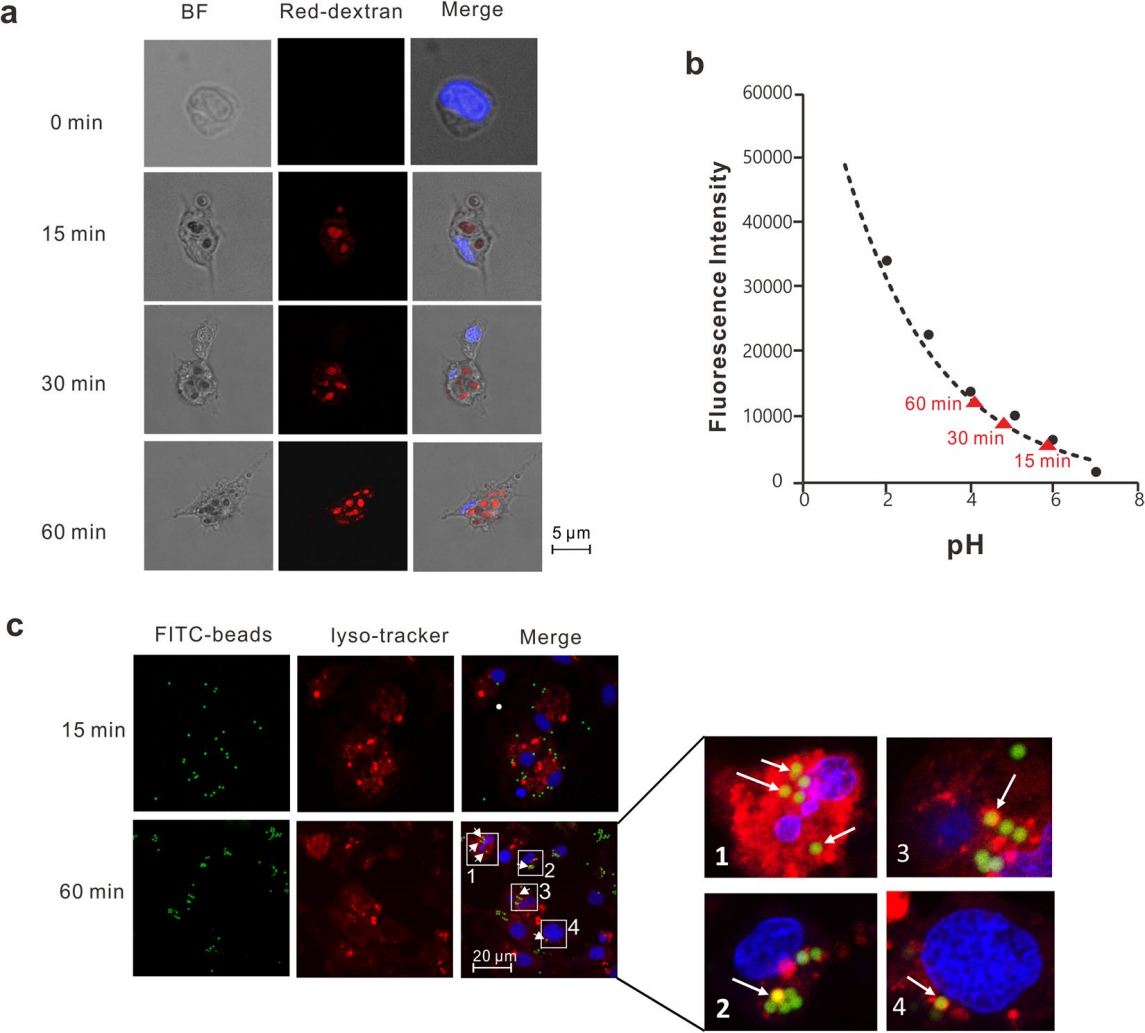
Changes in phagosomal pH following incubation with pH-sensitive fluorescent particles. (**a**) Oyster hemocytes were plated in glass-bottomed 35 mm dishes. pH-sensitive BioParticles for inducing phagocytosis were added at 1 mg/mL, followed by imaging using appropriate filters. DAPI was added to stain nuclei. BF: Bright Field; Red-dextran: pH-sensitive beads. (**b**) A calibration curve for quantification of pH values from the fluorescence intensities of pH sensitive beads. The red triangles represented the relative pH of oyster phagosomes after 15 min, 30 min, and 60 min phagocytosis respectively. (**c**) Green fluorescence represents the FITC-labeled beads. DAPI was used for nuclear DNA staining. Scale bar = 20 µm. LysoTracker excitation/emission: 577 nm/590 nm.

As part of an antibacterial response, hemocytes have evolved various weaponries for eradicating pathogenic invaders. Notable antimicrobial defense mechanisms include acidification of pathogen-containing phagosomes to a physiologically detrimental low pH. pH-sensitive dye conjugated beads are generally efficient pH sensors capable of rapidly and accurately reporting pH in an intracellular environment. They are non-fluorescence extracellularly, but fluoresce brightly in acidified phagosomes. To visualize the kinetics of hemocyte phagosomal acidification, images were sequentially acquired from 15 min to 60 min in the presence of pH-sensitive dye conjugates. Fluorescence images of pH-sensitive conjugates were merged with images of DAPI stain. Notably, during phagocytosis, the pH-dependent fluorescence could be visualized by microscopy. As shown in Fig. 1a, 15 min after challenge, phagocytosed dye conjugated beads reported relatively weak fluorescence, while maximal fluorescence was observed at 60 min. To quantify phagosomal acidification, a calibration curve was constructed as displayed in Fig. 1b. The average phagosomal pH was 5.9 at 15 min of phagocytosis and dropped to 4.7 at 30 min, while the pH continued acidification until 60 min of phagocytosis with pH of 3.8. Confocal microscopy analysis revealed that nascent phagosomes undergoes a process termed “phagosome maturation” to generate an acidified functional phagosome. Based on our imaging results, we defined 15 min as the time needed for forming early phagosomes and 60 min for late phagosomes.

### Characterization of phagosome-lysosome interactions

As lysosomes are dynamic organelles with functional implications in phagosome maturation, we next examined the interactions between lysosomes and phagosomes (Fig. 1c). Fifteen minutes after the initiation of phagocytosis, phagosomes enclosing FITC-beads had no discernable spatial overlap with lysosomes. However, fusion between phagosome and lysosome evidently occurred 60 min after the initiation of phagocytosis, suggesting the emergence of phagolysosomes. This result suggests that fusion with lysosomes conferred functionality to late phagosomes in oyster hemocytes.

### PPI (Protein-protein interaction) analysis of identified oyster phagosomal proteins

**Figure 2:**
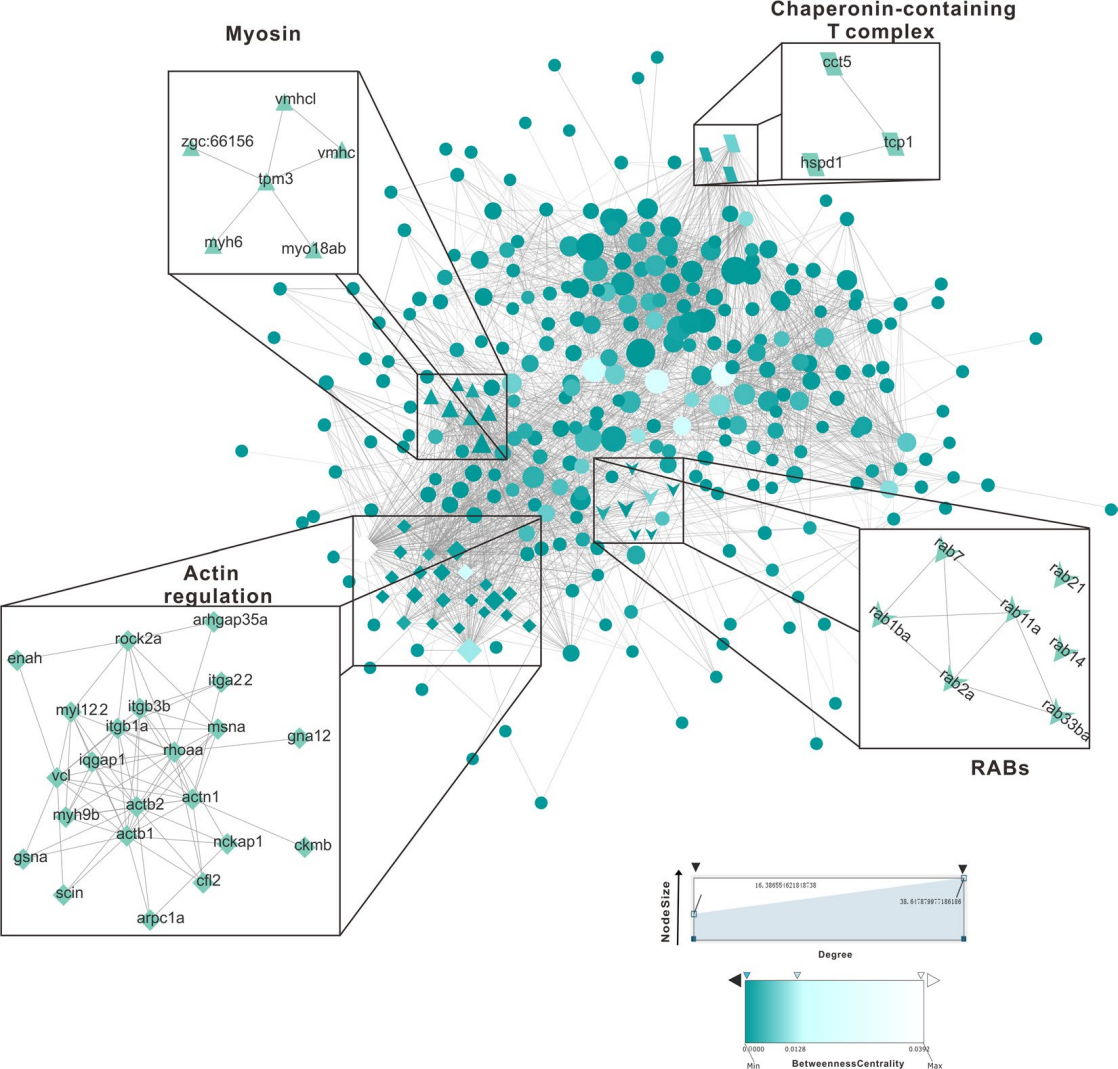
Phagosomal proteome network of oyster hemocytes. Phagosomal protein-protein interaction network of oyster hemocytes. The oyster phagosomal protein interactome is represented here graphically. Nodes represent proteins. Edges represent interactions between proteins. Sizes of a node are proportional to the degree of the node (a term defined as the amount of proteins that interact with the node), and colors of a node represent betweenness-centrality. Different shapes of a node represent 4 important protein groups including actin cytoskeletal regulation (diamond), myosin (triangle), chaperonin-containing T complex (parallelogram) and Rabs (arrows).

To delineate the dynamic details of oyster phagosomal proteins during phagocytosis, we mapped out a protein-protein interaction network based on datasets from MS analysis. All 410 proteins that identified from both early and late phogosomes were blasted against the *Danio rerio* protein database, and 351 of which were homologous with protein database and verified manually to ensure the accuracy of protein blast, before utilizing to construct an interactome comprising 1,427 interactions (edges) as shown in Fig. 2. For simplicity, the disconnected nodes were hidden in the presentation. Essentially, 4 sub-interactomes were depicted, with emphasis on proteins involved in actin cytoskeletal regulation, myosin, chaperonin-containing T complex and Rabs, in order to highlight their biological relationships in the phagosomal proteome. Information for these four protein groups were shown in Table S1 in Supplementary materials. Chaperonin-containing T complex, a chaperone protein that aids in the refolding of actin- and tubulin-based cytoskeletal components, was identified in the hemocyte phagosomal proteome, suggesting a role in homeostatic maintenance. Another noteworthy group of proteins identified is functionally related to regulation of actin cytoskeleton, containing some myosin.

Rab proteins, broadly known as Ras-related small guanosine‐5′-triphosphatases (GTPases), are mechanistically implicated in vesicle trafficking to target compartments. In our oyster phagosomal proteome, a total of seven different Rab proteins were identified, including Rab1, Rab2, Rab7, Rab11, Rab14, Rab21, and Rab33, suggesting the differences of an expanded pool of Rab proteins operating at the phagosomal interface between invertebrate and vertebrate. Among them, Rab1 and Rab14 were induced in the late phagosome, the relative expression level of which increased 1.31- and 1.30-fold, respectively.

### KEGG enrichment analysis of identified oyster phagosomal proteins

**Figure 3:**
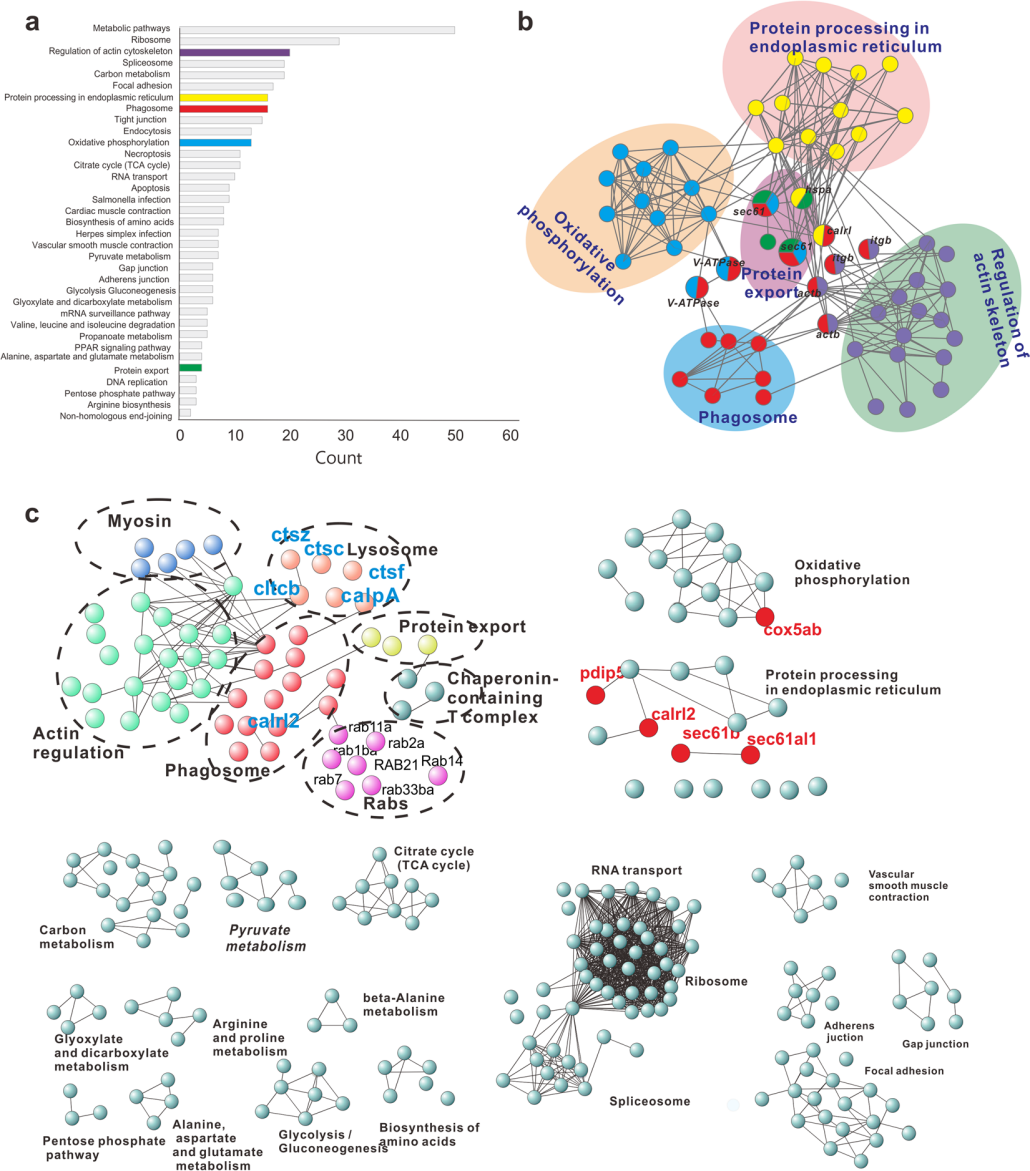
KEGG pathway analysis. (**a**) KEGG pathway enrichment analysis was summarized by a bubble chart. The x-axis shows the counts (number of a particular pathway), and the y-axis shows the pathway terms. Five optimized pathways were marked in color: purple, regulation of actin cytoskeleton; yellow, protein processing in the ER; red, phagosome; blue, oxidative phosphorylation; green, protein export. (**b**) Selected KEGG pathways closely related to phagosomes were used to construct a protein interactome, which includes these terms: regulation of actin cytoskeleton, phagosome, lysosome, protein export, Rabs and myosin. (**c**) Optimized networks were extracted according to KEGG pathways analyzed by STRING. Marker proteins of the ER and mitochondria are marked with red color.

In KEGG enrichment analysis, phagosomal proteins were found to be enriched in 35 signaling pathways (Fig. 3a). Metabolic pathway was the most conspicuously enriched signaling pathway containing 50 proteins. In addition, remarkable enrichment of phasosomal proteins was also observed in signaling pathways associated with ribosome function (29 proteins), regulation of actin cytoskeleton (20 proteins), splicesome (19 proteins), carbon metabolism (19 proteins), focal adhesion (17 proteins), phagosome (16 proteins), protein processing in the endoplasmic reticulum (16 proteins), oxidative phosphorylation (13 proteins), and so on. As phagosomes arise in part from the plasma membrane, it is predictable that some markers of the plasma membrane are shared by both compartments. These results suggested that the ER, mitochondria, and actin cytoskeleton work in coordination with biological processes within the oyster phagosome. Next, we constructed a PPI network integrating selected KEGG pathways (actin cytoskeleton, phagosome, protein processing in ER, protein export, oxidative phosphorylation). Proteins of interest were as shown in Fig. 3b, and in particular, proteins with overlapping roles among these pathways were marked, including V-ATPase (vacuolar-type H + -ATPase, which is responsible for acidifying a wide array of intracellular organelles and pumping protons across the plasma membrane), sec 61 (protein transport protein, as marker of the ER), calrl (calreticulin, a multifunctional protein that acts as a major Ca^2+^-binding or storage protein in the lumen of the endoplasmic reticulum), actb (beta-actin), itgb (integrin subunit beta), and hspa (heat shock 70 kDa protein).

Importantly, generation of an optimized phagosome network encompassing components of the *C. gigas* phagosomal proteome (Fig. 3c) helped elucidate the functional significance of proteins at work in the oyster phagosome. Proteins of phagosomal or lysosomal origin that likely occur as a component of phagocytosis were identified, which included cathepsin proteases (ctsc, ctsz, ctsf), calpain-A (calpA), and calreticulins (cltcb). In addition, proteins participating in oxidative phosphorylation and proteins processing in endoplasmic reticulum were noticeably enriched in the oyster phagosome, including the mitochondrial marker protein (cytochrome c oxidase, cox5ab), and the ER marker proteins (pdip5, calr2, sec61b, sec61al1). This suggests some functional involvement of mitochondria and ER in phagocytosis. Further analysis identified a number of proteins known to specifically form part of the ribosome and spliceosome. They are regarded as subcellular contaminants during fractionation experiments to isolate phagosomes, which may be co-purified along with phagosomes. Nonetheless, there is also a possibility that such proteins biochemically associate with phagosomes. Furthermore, proteins known to mediate metabolic processes (e.g. amino acid biosynthesis, TCA cycle, oxidative phosphorylation, etc.) were also identified. Given this fairly broad scope of identified proteins, further validation is needed to substantiate the relationship between these proteins in phagocytosis contexts and establish any new protein functions important to phagosomes.

### Characterization of interplay between phagosomes endoplasmic reticulum, and mitochondria

**Figure 4:**
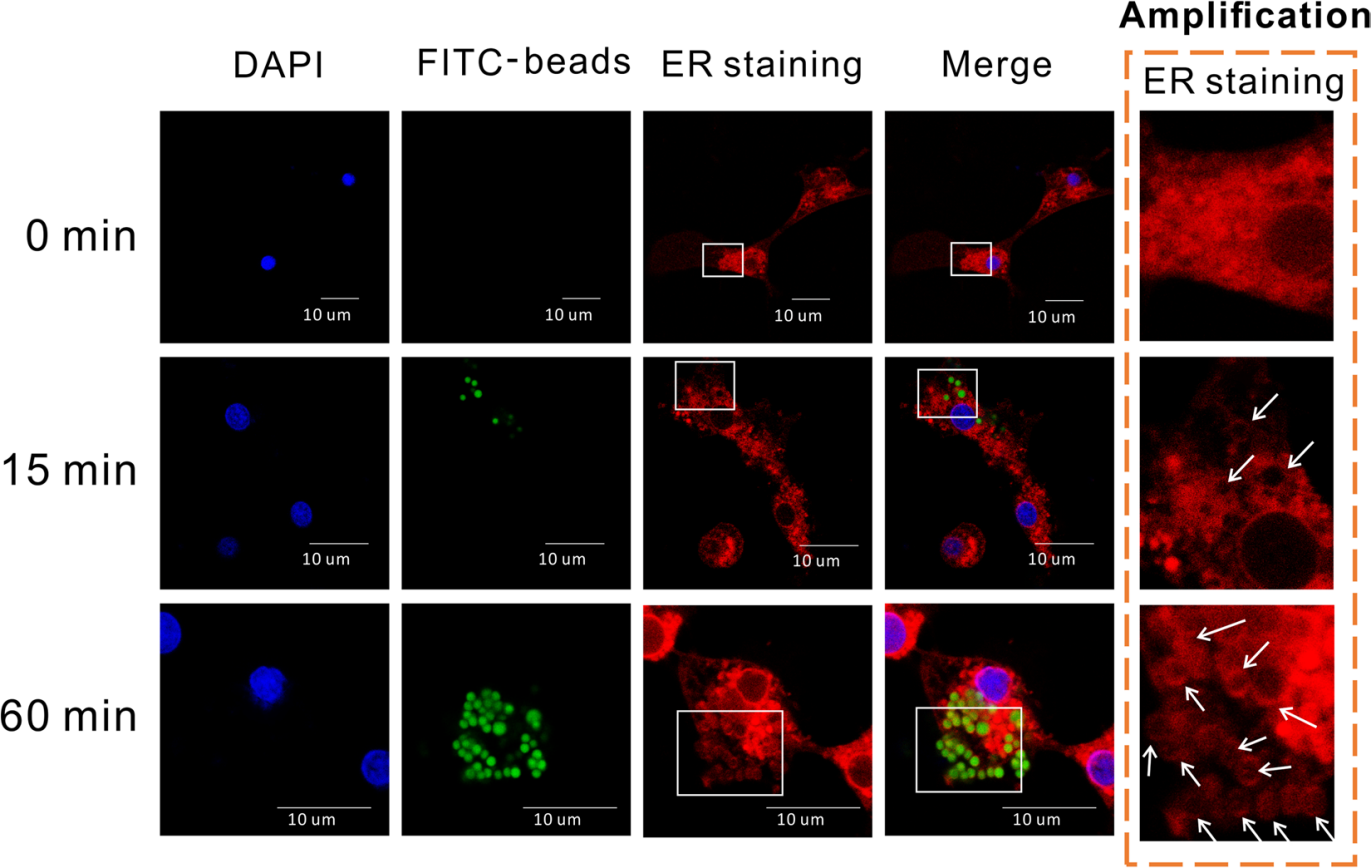
Study on organelle co-localization in oyster hemocytes stained with red detection regent in ER staining kit. Cells were stained with DAPI and ER staining. Green fluorescence represents FITC-labeled beads. Scale bar = 10 µm. ER staining excitation/emission: 579/599. White arrows indicate vesicles formed by ER.

As ER marker proteins had been identified in our hemocyte phagosomal proteome, we set out to explore the functional relationship between phagosomes and the ER in oyster hemocytes challenged with FITC-conjugated beads (Fig. 4). Kinetically, phagosomes were absent in hemocytes at 0 min, with the ER being predominantly located around the nucleus. Fifteen minutes after the initial challenge, FITC-conjugated beads began to fluoresce in early phagosomes. The ER remained mostly distributed in a perinuclear manner, while some of the FITC-beads became surrounded by the ER. Intriguingly, as the phagocytic process proceeded for 60 min, a large quantity of beads were engulfed into the hemocytes, and the ER also apparently clustered around the beads to form vesicles. These results strongly suggest an uncharacterized association between the ER and phagosomes in oyster hemocytes. We also examined any potential interactions between phagosomes and mitochondria during hemocyte phagocytosis at 15 min and 60 min as shown in Fig. S1 in Supplementary materials. No spatial overlap of the organelles was observed in either stage of phagocytosis, suggesting that no direct physical interactions exist between phagosomes and mitochondria.

### Comparative analysis on protein expression in phagosome formation and maturation

**Figure 5:**
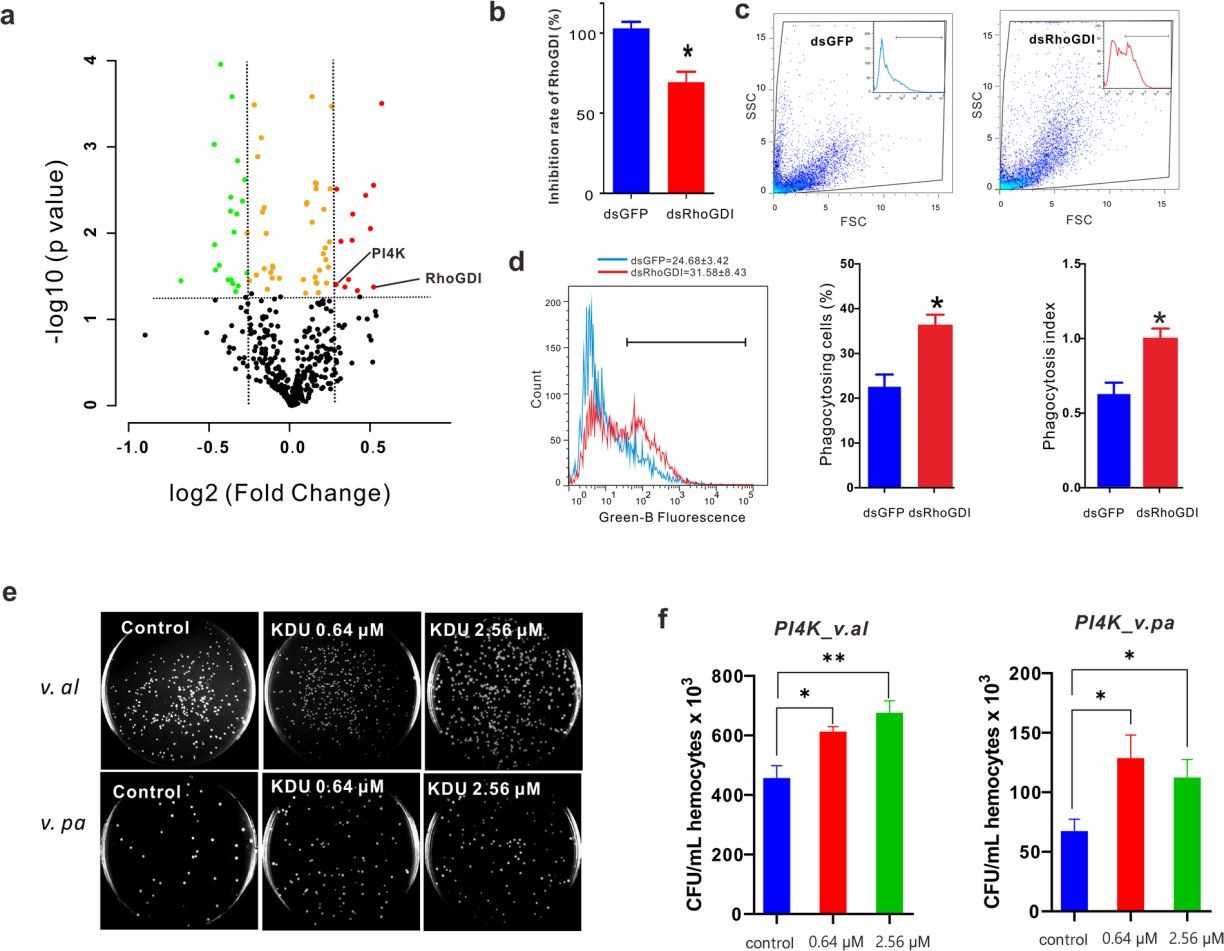
Differentially expressed proteins putatively involved in phagosome maturation with highlighted functions of *Cg*RhoGDI and CgPI4K. (**a**) Volcano plot displays the relationship between fold-changes and significance between early phagosomes and late phagosome. The y-axis is the -log10 of p-values and the x-axis is the difference in expression between two experimental groups as measured in log2 space. (**b**) Effects of RNAi on oyster *Cg*RhoGDI function. Expression of *Cg*RhoGDI was determined by RT-qPCR 3 days after 50 µg dsRNA injection. Inhibition rates of *Cg*RhoGDI were shown. (**c**) Flow cytometry assay was performed to gauge hemocyte phagocytosis. The left panel shows hemocytes of the dsGFP group, and the right panel shows the dsRhoGDI group. (**d**) The extent of phagocytosis (in percentage) of the *Cg*RhoGDI-depleted group was compared with that of the control group (dsGFP). The red line represents phagocytosis in the ds*Cg*RhoGDI group and the blue line represents phagocytosis in the dsGFP group. All data are presented as mean ± SEM (n = 3), with significance determined at *p < 0.05. (**e**) Representative images of agar plating results for *V. alginolyticus* and *V. parahemolyticus*. Treatment types are marked in the upper left corner of each image. KDU = PI4K inhibitor KDU691. (**f**) Statistical analysis on the effects of inhibitors on bacterial clearance by host cells. *p < 0.05, **p < 0.01.

In Fig. 5a, we used a volcano plot to summarize the magnitude, significance and variability in protein expression during phagosome formation and maturation in oyster hemocytes. Twelve identified proteins gave an ascending trend in protein expression level, while twenty proteins were down-regulated during phagosomes maturation. The significant protein expression was defined as p value less than 0.05, fold change greater than 1.2 and below 0.833, and Table 1 displayed the significantly differentially expressed proteins. Phosphatidylinositol 4-kinase (PI4K), which is tightly membrane-bound and therefore anticipated to be present on phagosome vesicles, were differentially expressed in late oyster phagosomes compared to that of early phagosomes. Additionally, according to analysis on protein expression in early phagosomes and late phagosomes, the expression levels of *Cg*RhoGDI in late phagosomes was 1.4-fold higher than that in early phagosomes.

**Table 1:** The information of DEPs (Differentially expressed proteins).

Prot_acc	Prot_name	Fold_Change	P_value
*Cg*K1QTP5	Solute carrier family 2, facilitated glucose transporter member 1	1.49	0.0355
*Cg*K1QCM0	Rho GDP-dissociation inhibitor 1	1.42	0.0473
*Cg*K1QTL3	Rho-associated protein kinase 2	1.42	0.0106
*Cg*K1PCN1	Uncharacterized protein	1.39	0.0178
CgK1R278	Tubulin beta chain	1.35	0.0322
*Cg*K1Q9P5	Mitochondrial-processing peptidase subunit beta	1.34	0.0299
*Cg*K1R2N0	Histone H4	1.31	0.0053
*Cg*K1QP10	Alpha-parvin	1.31	0.0063
*Cg*K1PW06	Filamin-C	1.29	0.0253
*Cg*K1PJW0	Talin-1	1.25	0.0337
*Cg*K1R4Z3	Malate dehydrogenase, mitochondrial	1.22	0.0379
*Cg*K1PPV5	Phosphatidylinositol 4-kinase type 2-beta	1.22	0.0146
*Cg*K1PI50	40 S ribosomal protein S26	0.83	0.0214
*Cg*K1R0L4	Sodium/potassium-transporting ATPase subunit alpha (EC 3.6.3.-)	0.83	0.0447
*Cg*K1QXS6	Heterogeneous nuclear ribonucleoprotein A2-like protein 1	0.82	0.0256
*Cg*K1QRZ3	40 S ribosomal protein S13	0.81	0.0405
*Cg*K1QFN1	60 S ribosomal protein L23	0.80	0.0348
*Cg*K1RU07	Glucose-repressible alcohol dehydrogenase transcriptional effector	0.80	0.0061
*Cg*K1R435	Splicing factor, arginine/serine-rich 4	0.80	0.0014
*Cg*K1QB93	DNA methyltransferase 1-associated protein 1	0.78	0.0280
*Cg*K1Q683	Coiled-coil domain-containing protein 47	0.78	0.0332
*Cg*Q70MP1	Ribosomal protein L5 (Fragment)	0.78	0.0114
*Cg*K1RA35	Splicing factor, arginine/serine-rich 7	0.78	0.0022
*Cg*K1Q6G5	60 S ribosomal protein L13	0.78	0.0014
*Cg*K1PEX0	GC-rich sequence DNA-binding factor-like protein	0.77	0.0362
*Cg*K1PHA1	Uncharacterized protein	0.74	0.0080
*Cg*K1R5G4	60 S ribosomal protein L31	0.74	0.0244
*Cg*K1PXU6	60S ribosomal protein L24	0.73	0.0179
*Cg*K1PYD7	60S ribosomal protein L28	0.72	0.0043
*Cg*K1QCE7	Uncharacterized protein	0.72	0.0069
*Cg*K1Q0L1	60S ribosomal protein L23a	0.63	0.0259
*Cg*K1QPB7	Uncharacterized protein	0.45	0.0119

### Functional analysis of RhoGDI and PI4K

To test its functional importance, we knocked down the expression of RhoGDI in oysters, with an interference efficiency of around 40% (Fig. 5b). Subsequently, we found an increase in the phagocytic ability of dsRhoGDI group of oyster hemocytes (Fig. 5c,d). Reduction in RhoGDI expression led to dissociation of GDP from Rho-GDP, yielding a Rho-GTP complex, which then promotes hemocyte phagocytosis against bacteria. Our results show that RhoGDI in oysters has an inhibitory effect on phagocytosis, which should be conserved within vertebrates.

In a parallel set of experiments, we examined the roles of PI4K as a regulator of phagocytosis in oyster hemocytes, using KDU691 as a PI4K specific inhibitor^26^. Assay was done by 15 min co-treatment of the inhibitor during a 15-min bacterial challenge in phagocytosis, followed by 15 min of gentamicin treatment for removing non-internalized bacteria. As expected, intracellular counts of *V. alginolyticus* and *V. parahemolyticus* increased in the presence of KDU691 (0.64 and 2.56 µM), as shown in Fig. 5e. The number of internalized bacteria in host cells, taken as a measure of phagocytosis efficiency, was markedly increased for groups treated with 0.64 µM or 2.56 µM KDU691 compared to that in the control group (Fig. 5f, Student’s t-test; statistical significance determined at p < 0.05). Specifically, the KDU691 groups had an intracellular viable count of *V. alginolyticus* at least 1.3 to 1.5 times higher than that of the untreated control (0.64 μM, p < 0.05; 2.56 μM, p < 0.01). Similarly, for *V. parahemolyticus* infections, bacterial CFU enumeration from host cells incubated with KDU691 had an 1.8-fold increase compared with the control group (0.64 μM, p < 0.05), thus confirming the impairment of bacterial killing via inhibition of PI4K inhibition in oyster hemocytes.

## Discussion

**Figure 6:**
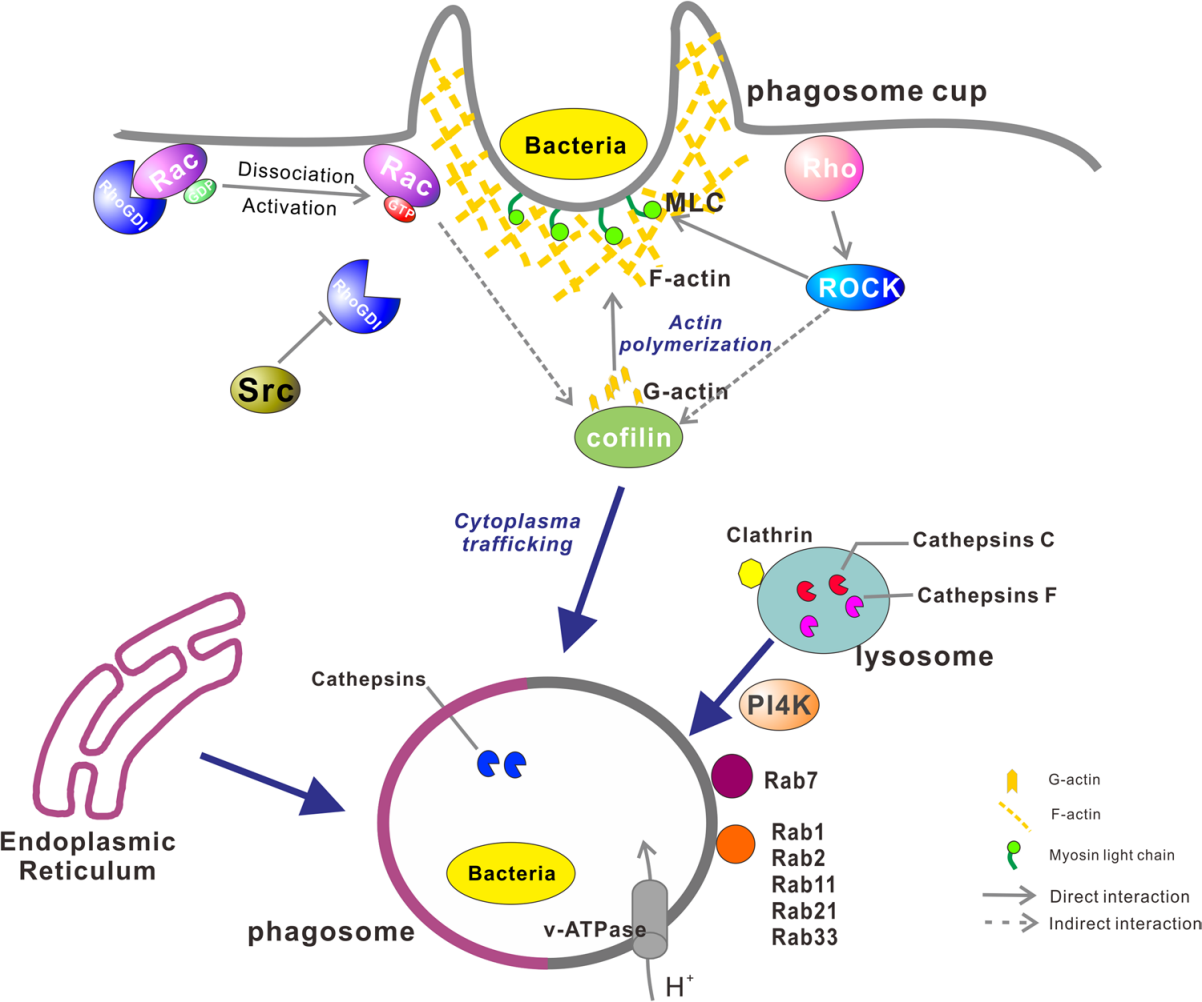
Conceptualization of key events and identified proteins in oyster phagosome formation and maturation. ROCK, Rho-associated protein kinase; RhoGDI, Rho GDP-dissociation inhibitor; Src, tyrosine-protein kinase; PI4K, phosphatidylinositol 4-kinase.

Phagocytosis is at once an ancient and physiologically fundamental mechanism for safeguarding host health against microbial invasion^27,28^. The dynamic and intricate nature of the protein machinery at work during phagocytosis necessitates ever better scope and resolution in proteomic analysis to provide a refined understanding of the process at the systems level. We herein applied an integrated approach to high-level purification of oyster phagosomes involving the deployment of differential centrifugation and density gradient sedimentation to render biological samples fully amenable to further molecular characterization of phagosomal proteins. Essentially, KEGG pathway analysis afforded a wealth of information on enriched proteins participating in the regulation of actin cytoskeleton. Dynamic actin cytoskeleton remodeling is particularly crucial for the precise execution and completion of efficient phagocytosis. Evidence has proved that cytoskeletal proteins, such as myosin, mediate a significant array of cellular processes, especially during the movement or remodeling of cell membranes for engulfing microbial invaders^29–31^. Therefore, it may be surmised that these proteins have some functional importance in regulating other under-examined cellular events in phagosome formation and maturation in *C. gigas*. Based on a dataset of identified phagosomal proteins from oyster hemocytes, we modeled the multifaceted process of phagosome formation and maturation in oysters as shown in Fig. 6. The first step involves disrupting the membrane-associated cortical cytoskeleton, which potentially has two functions. One is to release G-actin monomers for building new filaments, while cofilin (*Cg*K1PYB0) can regenerate the actin pool by incising and depolymerizing F-actin filaments. The second is to promote the nucleation process of actin filaments, which in turn initiates the polymerization of F-actin. In “resting” phagocytes, the Rho family of proteins binds to GDP, and the promotion of actin filament formation requires the active forms (GTP-bound). Rho members are implicated in myosin light chain (MLC) phosphorylation, which drives interaction between actin and myosin, and inhibits bacterial infection^32,33^. It has been reported that RhoGDI is a negative regulator of Rho family GTPases, inhibiting the movement of actin cytoskeleton^34^. In oyster hemocytes, we further demonstrated that *Cg*RhoGDI can dampen hemocytes’ phagocytic ability to engulf bacteria, through RhoGDI inhibition of Rho activation which likely checks phagocyte motility and formation of the phagosome cup.

In addition to the conserved mechanisms of actin cytoskeleton remodeling for forming phagosomes, there has been corroborative evidence that maturing phagosomes are capable of luminal acidification, culminating in the fusion with lysosomes to yield immunologically professional phagolysosomes, which are indispensable in immune processing and pathogen clearance^35^. Consistent with this, fluorescence enhancement of pH reporters in acidifying oyster phagsosomes likely reflected a drop in pH, suggesting that rapid phagosome acidification was a highly conserved function among species from marine invertebrates to terrestrial vertebrates. While some aspects of phagosome acidification have been explored in detail in mammalian receptor-mediated signaling pathways^31,36,37^, three different types of membrane receptor integrins (*Cg*K1QAH5, *Cg*K1QIL4, and *Cg*Q95P95) were also identified in our oyster phagosomal proteomic, which has been indicated to be involved in regulating phagosome maturation through Rac expression^37^. Notably, a chloride (Cl^−^) intracellular channel (*Cg*K1R973) was found in the oyster phagosomal proteome, while Cl^−^ is associated with acidification. Clathrin-containing coated vesicles catalyze ATP-dependent proton translocation, wherein chloride serves as a counter-ion for balancing electrogenic proton pumping, participates in phagosomal pH control, and helps sustain bacterial killing^38–40^. Previously, phagosomal membrane proteins and vesicular transport proteins have also been implicated in phagocytic functions. These include the V-type proton ATPase (*Cg*K1QKL8 and *Cg*K1Q9V3) we identified in oyster phagosome, which executes proton (H^+^) transport, regulates intraphagosomal pH, and thus promotes acidification and maturation of phagosomes^6,41,42^. In addition, a host of proteins contributed to the highly regulated process of phagosome maturation, with modulatory roles in vesicular trafficking and membrane fusion. Among differentially expressed proteins, PI4K was highly expressed in the late phagosomes, suggesting some role in the terminal removal of foreign particles or microorganisms. Phosphatidylinositol phosphates PI(4)P is involved in fusion of phagosomes with various endocytic compartments^43^, which can be generated by PI4K. In our own results, the ability to kill bacteria by host cells was blunted by the PI4K inhibitor KDU691, supporting the functional relevance of PI4K in phagosome maturation and pathogen clearance. Rab proteins, which are key regulators of membrane traffic, have been implicated in diverse processes wherein the transport vesicles traffic to their target compartments^44,45^. In this study, members of the Rab protein family (Rab1, Rab2, Rab7, Rab11, Rab14, Rab21, and Rab33) were identified as active players during phagocytosis, raising the possibility that they had distinct regulatory roles in early phagosomes and late phagosomes. Oyster phagosomes harbored similar processes of formation and maturation to vertebrates, suggesting that phagocytosis was a very ancient and conserved strategy of immune system.

Some of the most conspicuous protein expression differences in our proteomic analysis was observed in Rab proteins, which were well-documented key regulators of membrane traffic in biosynthetic and endocytic pathways. Over 60 different Rab proteins within the cells is involved in regulating specific steps of membrane trafficking^46^. Previously, Rab5 was reported to be predominantly involved in the formation of clathrin-coated vesicles^47^ and the fusion of coated vesicles with sorting vesicles^48^. Rab7 is localized to late endosomes and to lysosomes, and is responsible for orchestrating continuous fusion events between late phagosomes and lysosomes^49^. In addition, it has also implicated in transport vesicles from early to late endosomes^49,50^. In our current study, however, our oyster phagosome proteomics identified seven Rabs (Rab1, Rab2, Rab7, Rab11, Rab14, RaB21, and Rab33) proteins, but not Rab5. Functional components of the phagocytosis machinery seems to display variations, as invertebrates and vertebrates evolve in their separate niches. Understandably, disparity also may exist in the corresponding molecular mechanisms between invertebrates and vertebrates. For example, the expression level of Rab7 had no different in the late phagosome of oyster, which is distinguish in vertebrate. But, Rab14 activity regulates phagsosome maturation by recruiting to Rab7 during pathogen infection^51,52^, and the up-regulation of Rab14 in the late oyster phagosome corresponds to these previous studies. Similar but distinct mechanisms may be operating in evolutionarily more ancient species such as marine invertebrates. The detailed molecular function of distinct Rabs in the phagosomal proteome of oyster hemocytes is yet to be clarified by careful experimentation.

After nascent phagosomes take form on the cell surface, they ripen into a mature state through a complex maturation process, requiring extensive interactions with various cellular organelles. A wealth of literature over the past decade have shown that some of the characteristic functions of phagosomes occur in specific membrane domains. Intriguingly, a variety of ER-related proteins were also identified in both early phagosomes and late phagosomes in Pacific oysters, among which calnexin, sec61 subunit, and protein disulfide-isomerases are known as maker proteins of ER. Functionally, the ER is an intricate membrane structure devoted to the biosynthesis of proteins, sugars and lipids and intracellular transport. Previously, quantitative proteomics showed that the percentage of membranes contributed by plasma membranes and ER membranes to phagosomes at 10 min after internalization was approximately at a ratio of 50:1^53^, suggesting that the contribution of ER lipid components to phagosomes is negligible. However, phagosomes were also shown to fuse with the ER shortly after exogenous substances were phagocytosed in dendritic cells^54^. At the molecular level, some ER maker proteins, such as sec61/sec62, have been found in isolated phagosomes, supporting to the idea that phagosomes may physically interact with the ER during phagosome formation. In contrast, calnexin of the ER decreases progressively with the maturation of phagosomes^55^. Recent findings by quantitative proteomics methods have advanced the proposition that the ER supplies a subset of the phagosome membranes but only contributes moderately to the early phagosome proteome (approximately 20%)^56^. Even though many studies have attempted to elaborate on the plausible function of the ER in phagocytosis^57,58^, mechanistic links between the ER to phagosomes have remained at controversial in this field. Our study provided supporting evidence that ER provided certain membrane part during the formation and maturation of hemocyte phagosomes in an invertebrate, the Pacific oysters, in addition to the contribution of plasma membrane, thus implicating the ER as a potentially important organelle regulating phagocytic processes in invertebrates. Our results for confocal imaging confirmed that the ER aggregated and formed vesicles around FITC-conjugated beads during late phagocytosis, supporting the hypothesis that phagocytic particles slide into the ER via an opening formed at the base of the phagocytic cup^14,55,59^.

Proteomic analysis is a powerful approach to provide an objective, accurate view of the dynamic proteome landscape defining phagocytosis in invertebrates and vertebrates alike. Our quantitative proteomic investigation indicated that distinct molecular mechanisms existed to govern the signaling events in phagosome formation and maturation in oyster hemocytes. Of note, the small GTPase negative regulator *Cg*RhoGDI served to inhibit phagosome formation, while the versatile kinase PI4K was required for phagosome maturation. Proteomic and live cell imaging evidence supported the notion of dynamic engagement between the ER and phagosomes in phagocytosis. Collectively, our work has furnished new molecular details for a more refined model to predict events in phagosome formation and maturation in oyster hemocytes. In order to dissect the origins and vital roles of phagosomes in immunity and diseases, further efforts via systems-level investigation on subcellular proteomes and interactomes are warranted.

## Materials and Methods

### Oyster collection and maintenance

Pacific oysters, *Crassostrea gigas* (two years old with an average 100 mm shell length), were obtained from Qingdao, Shandong Province, China, and maintained at 22–25 °C in tanks with re-circulating seawater before experiments. Treatment-naïve and pathogen-free oysters were chosen for experiments, independently of their genetic background. Oysters were fed twice daily on *Tetraselmis suecica* and *Isochrysis galbana*. They were held for two weeks prior to experimentation.

### Phagosome isolation, protein purification and mass spectrometry

**Figure 7:**
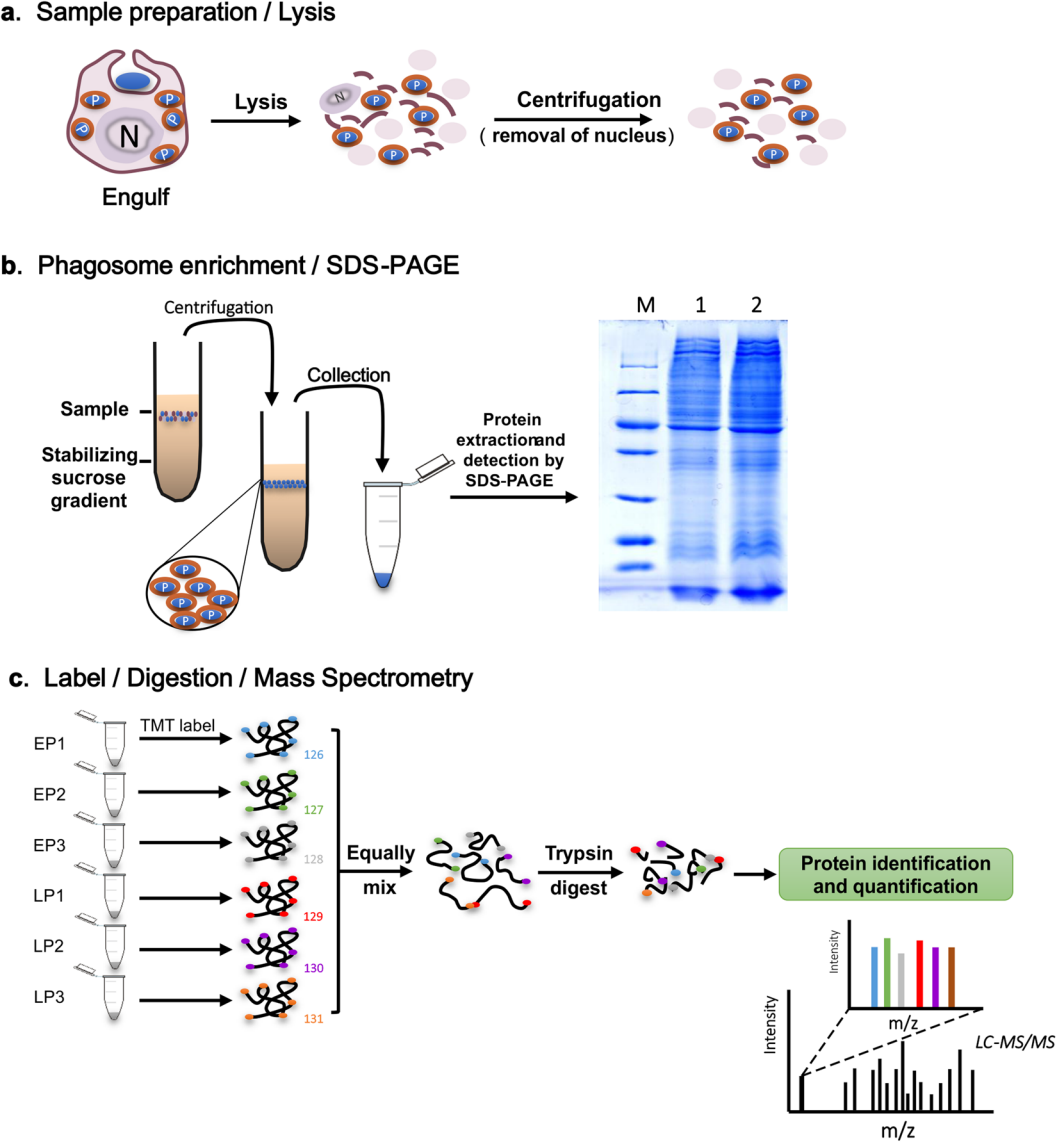
Fractionation of oyster hemocyte phagosomes for proteomic analysis. (**a**) Oyster hemocytes were incubated with 0.8 µm blue-dyed latex beads (Sigma) for 15 min and 60 min, respectively. Subsequently, they were lysed to liberate the phagosomes. (**b**) Phagosomes were isolated on a sucrose gradient and detected by SDS-PAGE.

To identify phagosome proteins of Pacific oysters, we undertook comparative MS analysis on phagosome extracts from two groups with different time points in endocytosis (15 min and 60 min), corresponding to early phagosomes and late phagosomes. Figure 7 illustrates the workflow for phagosome isolation and proteomic analysis. Three biological replicates per group (each in 2 technical replicates) were used. Latex beads have been generally used in the induction and isolation of phagosomes in cells, which has been reported for decade years^8,53,60–62^. We applied these methods for phagosome isolation with some modifications^5^. Briefly, 0.8 µm blue latex beads (L1273, Sigma) were washed twice to remove detergent and sodium, re-suspended in Sorensen buffer (SB) (15 mM KH_2_PO_4_, 2 mM Na_2_HPO_4_, pH 6.0) and kept on ice. Oyster hemocytes were collected and maintained on ice. Latex beads were then added to the hemocytes and mixed by inverting tubes for internalization into the cells. Phagosomes formed upon internalization of blue latex beads for 15 min and 60 min were referred to the early phagosomes and late phagosomes, respectively. Cells were subsequently collected and lysed in homogenization buffer as per the methods described by Desjardins *et al*.^60^. Unbroken cells were separated by centrifugation at 1,200 rpm for 5 min at 4 °C and the supernatant containing phagosomes were subsequently isolated by flotation on a sucrose gradient (10%-25%-35%-40%-62%) for yielding enriched phagosomal protein extracts.

Next, protein extracts were visualized by SDS-PAGE as detectable bands. Tandem mass tags (TMTs) was used as isotopomer labels for the accurate quantification of peptides and proteins. These tags and their analytical methods allowed peptides from different samples to be identified by their relative abundance^63^. Equal amounts of proteins of labeled samples were mixed, followed by trypsin digestion. Liquid chromatography-tandem mass spectrometry (LC-MS/MS) was then performed to identify phagosomal proteins and compare their abundance across biological replicates (n = 3) of early phagosomes and late phagosomes in Pacific oysters.

In detail, the dried peptides TMT-labeled peptides were prefractionated by offline Strong Cation Exchange (SCX) chromatography via a Poly SULFOETHYL column (200 × 4.6 mm, 5 μm particle size, 200-Å pore size) (PolyLC, Columbia, MD) using a Waters 2695 HPLC. Reconstituted dried peptide fractions were analyzed with a LTQ-Orbitrap Elite (Thermo Fisher, Bremen, Germany) coupled with an Easy-nLC (Thermo Fisher, Bremen, Germany). Peptides were separated by a C18 capillary column (50 μm × 15 cm, packed with Acclaim PepMap RSLC C18, 2 μm, 100 Å, nanoViper, Thermo Scientific). Finally, a full mass spectrometry (MS) scan (350–1600 m/z range) was acquired at a resolution of 60,000 in the positive charge mode. The five most abundant precursors with +2, +3 or +4 charges and over a minimum signal threshold of 500.0 were selected for fragmentation using a collision-induced dissociation (CID)/ high-energy collision-induced dissociation (HCD) dual scan approach.

### Mass spectrometry data processing

Raw data inclusive of two technical replicates were converted into .mgf files by using Proteome Discovery 1.3.0.339 (Thermo Finnigan). A python script was applied to filter low-quality scans and separate the data in to HCD and CID files^64^. Then, the separated .mgf files were searched against *C. gigas* a genomic database with 55,358 amino acid sequences including both target and decoy sequences in Mascot version 2.3.2 (Matrix Sciences). Search parameters used for HCD files were based on previous works^65^, specifically: variable modification for oxidation of methionine and deamidation of glutamine and asparagine; fixed modification for carbamidomethyl of cysteine; maximum missed cleavage of trypsin set at two; 5 ppm for peptide mass tolerance and 20 mmu for MS/MS tolerance. For CID files, search parameters were similar to those for HCD except that the MS/MS tolerance was set as 0.6 Da. Proteins meeting the following criteria were retained as valid identification: matched peptides with an ion score ≥ 27 (corresponding to 95% confidence level); peptide length > 7 amino acids; FDR < 0.01. The mass spectrometry proteomics data have been deposited to the ProteomeXchange Consortium via the PRIDE^66–68^ partner repository with the dataset identifier PXD015810.

### pH dependent fluorescence curve

Phagosomal pH was quantified by measuring fluorescence intensity of pH-sensitive BioParticles (P35361, Thermo Fisher, USA), which has reported previously^69^. Briefly, the pH dependent fluorescence curve was acquired by immersing pH-sensitive beads in standard buffers with serial pH gradient (pH 2–7). The pH-sensitive beads from buffers of different pH were loaded into confocal dishes. The image was obtained by Leica LP8X confocal microscope (Germany) and the fluorescence intensities were calculated using the LAS X Small 3.3.0 software. The data was analyzed and visualized via Graphpad Prism 8. The final quantification was averaged from four replications, and each replication contains 30 fluorescence beads.

### Measurement of phagosomal acidification by fluorescence microscopy

Oyster hemocytes were cultured in confocal dishes with a suspension at a density of 10^5^ cells/mL and a volume of 500 µL per sample. After 15 min of incubation, cells were infected for 15 min, 30 min, and 60 min by adding pH-sensitive BioParticles (P35361, Thermo Fisher, USA) at a concentration of 1 mg/mL. Cells were then washed three times to remove extracellular particles. After phagocytosis, paraformaldehyde (4%, cold) was used to terminate the phagocytic process and fix cells for 15 min. Cells were washed three times with PBS, followed by DAPI (D9542, Sigma, USA) staining of nuclei for 5 min. For each time point, several pairs of images (generally four) were acquired with appropriate filters by Leica LP8X confocal microscopy. After acquisition, images were analyzed by Image Pro Plus software and corresponding fluorescence intensity data by GraphPad5 software.

### Analysis of protein-protein interaction networks

STRING server (http://string.embl.de) was used to predict interacting partners in protein-protein interactions. STRING database leverages a combination of prediction algorithms and integration of other information (e.g. neighborhood, transferred neighborhood, gene fusion, co-occurrence, co-expression, experiments, databases, text mining). We compared these protein-protein interactions by using the protein databases of *Danio rerio* and *Drosophila melanogaster*, which are species considered evolutionarily relevant to oysters. Further, *Danio rerio* protein database was found to be more suitable for constructing protein interactomes of oyster phagosomes, based on the number and similarity of aligned proteins. Networks were constructed at a high confidence level (0.700) allowing all active prediction methods. Disconnected nodes were hidden. The entire network was available for interactive visualization of protein interactions in Cytoscape session file. All the additional network presented in the paper were constructed using STRING.

Protein IDs used for optimized-networks were listed in Supplementary Table S1. In terms of function within these networks, 22 proteins were assigned to “actin regulation”, 6 to “myosin”, 3 to “chaperonin-containing T complex” and 7 to “Rabs proteins.”

### KEGG pathway enrichment analysis

Pathway enrichment analysis is useful for determining the main metabolic pathways and signaling pathways implicating identified proteins. In this study, KEGG pathway analysis was performed^70–72^, whose results were represented as a histogram. To further explore interactions among KEGG pathways closely related to phagosomes, PPI network analysis was performed with STRING database. Different colors were assigned to different selected KEGG pathways, including regulation of actin cytoskeleton, phagosomes, protein processing in the ER, protein export, and oxidative phosphorylation. Accordingly, an optimized protein network was built.

### Confocal microscopic validation of organellar interactions

For this experiment, oyster hemocytes were plated on confocal dishes and were maintained in culture medium for 15 min before addition of endoplasma reticulum-, and lysosome-selective dyes. After incubation, FITC-labeled beads were added to hemocytes and incubated for specified durations (15 min and 60 min). To trace lysosomes, cells were incubated with LysoTracker (L7528, Thermo Fisher, USA). Upon labeling, cells was washed three times and fixed with acetaldehyde. Finally, cells were co-stained with DAPI (300 nM) for 5 min. Images were observed with a Leica LP8X confocal microscope.

For ER staining experiments, the CytoPainter ER staining kit (red fluorescence; ab139482, Abcam, USA) was used. After plating cells for 15 min, FITC-labeled beads were added to hemocytes for appropriate durations of incubation (15 min and 60 min). Then, cells were washed with assay buffer, followed by fixing with freshly prepared 3.7% formaldehyde solution at 37 °C for 10 min. After fixation, ER staining was performed with the red fluorescence detection reagent and Hoechst 33342 nuclear dye, which was pre-diluted in 1 mL assay buffer. Finally, images were acquired with a Leica LP8X confocal fluorescence microscope.

### Data analysis

Statistical difference was calculated by Student’s t-test for triplicated data of early phagosomes and late phagosomes and only groups with p-value < 0.05 were considered statistically significant. In addition, averages of triplicate values for early and late phagosomes were compared, and proteins with fold changes greater than 1.2 or below 0.833 were considered differentially expressed. Finally, we used a volcano plot to delineate changes in protein expression. Volcano plot was constructed by using the -log of p-values on the y-axis (base 10) where data points with low p-values (highly significant) appeared on top of the plot, and values of log2 of fold changes for early phagosome and late phagosome groups were plotted on the x-axis.

### RNAi assay

To clarify the roles of *Cg*RhoGDI in phagocytosis of oyster hemocytes, this gene was knocked down *in vivo* via dsRNA-mediated RNA interference. Primers used to synthesize dsRNA are as shown in Table 2. *Cg*RhoGDI and a GFP cDNA fragment (negative control) were amplified with primer pairs with T7 promoter overhangs in the Primega RiboMAXTM Express RNAi System. PCR products were used as templates to synthesize dsRNA according to the manufacturer’s instructions. For this experiment, 20 oysters were randomly divided into 2 groups housed in 2 tanks: *Cg*RhoGDI-depleted group and control group. Each oyster was injected with 50 µg dsRNA and three individuals from each group were randomly selected for the collection of hemocytes. RNAi efficiency was determined by RT-PCR.

**Table 2:** Primers used in this study.

Name of Primers	Sequence (5′ - 3′)	
*Cg*RhoGDI-F	AGGATGAAAGTCTACGCAAAT	PCR of CgRhoGDI
*Cg*RhoGDI-R	GTATGACCCTCGCACCAG	
Q*Cg*RhoGDI-F	GGAAGTTATGGACCCAAGGAGAC	qPCR of CgRhoGDI
Q*Cg*RhoGDI-R	GATGGAGTTGCGGTCGTCA	
*Cg*RhoGDI-dsF	GGATCCTAATACGACTCACTATAGGAGGATGAAAGTCTACGCAAAT	PCR of CgRhoGDI dsRNA
*Cg*RhoGDI-dsR	GGATCCTAATACGACTCACTATAGGGTATGACCCTCGCACCAG	
GADPH-F	GGATTGGCGTGGTGGTAGAG	qPCR of GADPH
GADPH-R	GTATGATGCCCCTTTGTTGAGTC	

### Phagocytosis assay

*Escherichia coli* DH5α was transformed with a pFPV25.1 plasmid. Valid transformants containing this plasmid could emit green fluorescence. Transformed *E. coli* was cultured overnight, harvested and washed three times with cold PBS. Then, bacteria were added to hemocytes cultured in a 24-well plate for 15 min, in a 50:1 ratio. Next, hemocytes were washed three times with Tris buffer (pH 8.0, 50 mM) and re-suspended in PBS supplemented with 15% EDTA. Finally, flow cytometry assay was performed to quantify phagocytosis-related fluorescence in oyster hemocytes. The gate was applied to identify a specific population, in this case oyster hemocytes. Hemocyte phagocytosis was monitored by using at least 10,000 event per sample. Data were analyzed with the FlowJo software.

### Bacteria clearance assay

First, oyster hemocytes were harvested from the pericardial cavity, and seeded into a 24-well plate for 15 min. Ten microliter of bacteria (*Vibrio alginolyticus* ZJ51 and *Vibrio parahaemolyticus* E151) at a final density of 2.5 × 10^7^ CFU/mL were added to host cells (5 × 10^5^ hemocytes/mL) in plasma, which were incubated at room temperature for 15 min. Upon establishment of phagocytosis (15 min), all samples were washed twice to remove non-phagocytic bacteria and further incubated with gentamicin (5 mg/mL) for 15 min to eliminate extracellular live bacteria. This was followed by addition of inhibitors in appropriate concentrations (KDU691 at 0.65 and 2.56 µM) for 15 min. Further determination of intracellular live bacteria counts was done 15 min later and the host cells were lysed with 1 mL of 0.1% X-triton per sample. One hundred microliter of the lysate was plated in LB (Luria-Bertani) solid medium. Subsequently, bacteria colonies were enumerated 16 h post-inoculation with the aid of Image Pro software.

### Safety statement

Experiments in this study were followed standard biosecurity and institutional safety procedures.

### Ethics statement

Experiments in this study were conducted with approval from Experimental Animal. Ethics Committee, South China Sea Institute of Oceanology, Chinese Academy of Sciences, China.

## Supplementary information

Supplementary information.

Supplementary information 2.
