# Supplementary material for: Hemocyte phagosomal proteome is dynamically shaped by cytoskeleton remodeling and interorganellar communication with endoplasmic reticulum during phagocytosis in a marine invertebrate, Crassostrea gigas
Source: Sci Rep. 2020 Apr 20;10:6577. doi: 10.1038/s41598-020-63676-3 (PMC7171069; doi:10.1038/s41598-020-63676-3)
Supplement: Supplementary file 1 — Supplementary information. [file 41598_2020_63676_MOESM1_ESM.pdf]

Hemocyte phagosomal proteome is dynamically shaped by cytoskeleton remodeling and interorganellar communication with endoplasmic reticulum during phagocytosis in a marine invertebrate, *Crassostrea gigas*

Fan Mao [1, 2, 3], Huawei Mu [4], Nai-Kei Wong [5], Kunna Liu [1, 2, 3], Jingchen Song [6], Jianwen Qiu [7], Yue Lin [1, 2, 3], Xiangyu Zhang [1, 2, 3], Duo Xu [1, 2, 3], Zhiming Xiang [1, 2, 3], Jun Li [1, 2, 3], Yang Zhang [1, 2, 3*], Ziniu Yu [1, 2, 3 *]

[1] CAS Key Laboratory of Tropical Marine Bio-resources and Ecology and Guangdong Provincial Key Laboratory of Applied Marine Biology, South China Sea Institute of Oceanology, Chinese Academy of Science. Guangzhou, China;

[2] Innovation Academy of South China Sea Ecology and Environmental Engineering, Chinese Academy of Sciences, ISEE, CAS. Guangzhou, China;

[3] Southern Marine Science and Engineering Guangdong Laboratory (Guangzhou). Guangzhou, China;

[4] School of Life Sciences, University of Science and Technology of China. Hefei, China;

[5] Department of Infectious Diseases, Shenzhen Third People's Hospital, The Second Hospital Affiliated to Southern University of Science and Technology. Shenzhen, China;

[6] College of Oceanology, South China Agricultural University. Guangzhou, China;

[7] Croucher Institute for Environmental Sciences and the Department of Biology, Hong Kong Baptist University. Hong Kong, China.

Running title: *Phagosomal proteomics in C.gigas*

[*]**Correspondence:** South China Sea Institute of Oceanology, Chinese Academy of Sciences, 164 West Xingang Road, Guangzhou 510301, China.

Tel. /fax: +86 20 8910 2507.

E-mail address: yzhang@scsio.ac.cn (Y. Zhang).

E-mail address: carlzyu@scsio.ac.cn (Z. Yu)

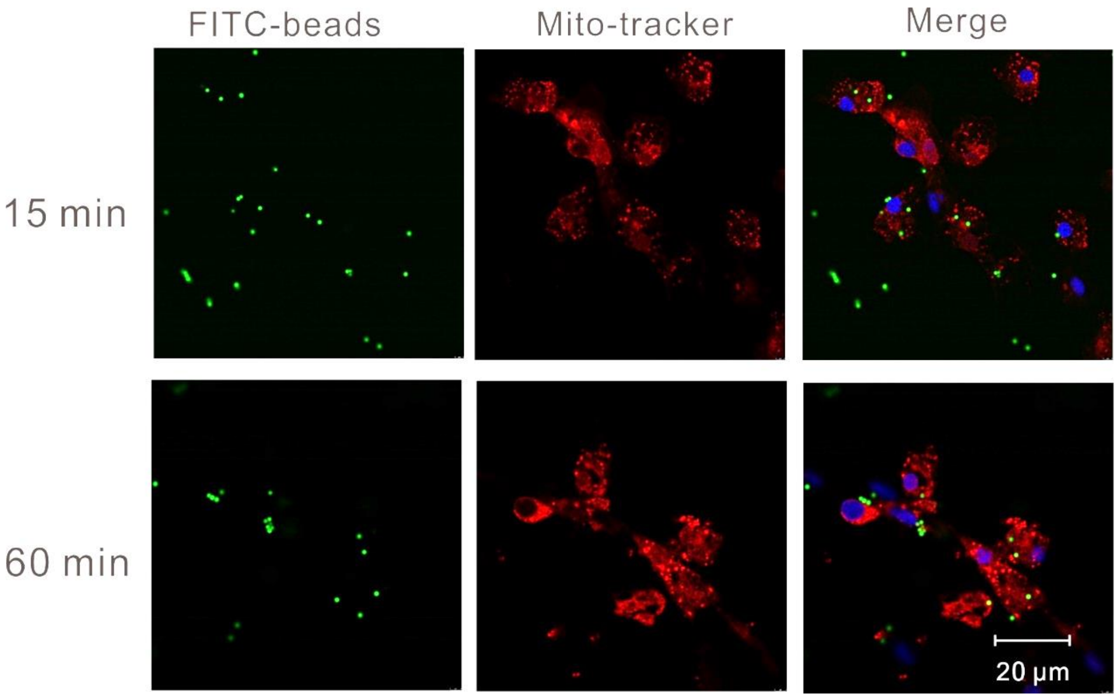

Figure S1 Confocal images of oyster hemocytes staining with mito tracker at 63× using zoom in. To trace mitochondria, cells were incubated with MitoTracker (M7512, Thermo Fisher, USA). Upon labeling, cells was washed three times and fixed with acetaldehyde. The green fluorescence is the FITC labeled beads. Scale bar = 20 μm. MitoTracker Excitation/Emission: 579/599.
