# Supplementary material for: Hemocyte phagosomal proteome is dynamically shaped by cytoskeleton remodeling and interorganellar communication with endoplasmic reticulum during phagocytosis in a marine invertebrate, Crassostrea gigas
Source: Sci Rep. 2020 Apr 20;10:6577. doi: 10.1038/s41598-020-63676-3 (PMC7171069; doi:10.1038/s41598-020-63676-3)
Supplement: Supplementary file 2 — Supplementary information 2. [file 41598_2020_63676_MOESM2_ESM.docx]

**Table S1** Protein IDs used for the optimized-networks

| **Group name** | **STRING ID** | **Protein ID** | **Gene name** |
| --- | --- | --- | --- |
| **Actin regulation** | nckap1 | CgK1RCN5 | Nck-associated protein 1 |
|  | myh9b | CgK1QDH9 | Myosin-11 |
|  | arpc1a | CgK1R488 | Actin-related protein 2/3 complex subunit 1A |
|  | ckmb | CgK1PLF9 | Arginine kinase |
|  | cfl2 | CgK1PYB0 | Cofilin |
|  | myl12.2 | CgK1Q122 | Myosin regulatory light chain sqh |
|  | iqgap1 | CgK1PNK5 | Ras GTPase-activating-like protein IQGAP1 |
|  | arhgap35a | CgK1S493 | Uncharacterized protein |
|  | gna12 | CgK1RBW4 | Guanine nucleotide-binding protein subunit alpha-12 |
|  | rock2a | CgK1QTL3 | Rho-associated protein kinase 2 |
|  | enah | CgK1QY97 | Vasodilator-stimulated phosphoprotein |
|  | gsna | CgK1PE57 | Severin |
|  | scin | CgK1PCV0 | Severin |
|  | msna | CgK1PUJ1 | Radixin |
|  | itga2.2 | CgK1QIL4 | Integrin alpha-4 |
|  | itgb3b | CgK1QAH5 | Integrin beta |
|  | itgb1a | CgQ95P95 | Integrin beta |
|  | vcl | CgK1P339 | Vinculin |
|  | rhoaa | CgK1QVS0 | Ras-like GTP-binding protein Rho1 |
|  | actn1kca | CgK1RH58 | Alpha-actinin, sarcomeric |
|  | actb2 | CgK1RA57 | Actin |
|  | actb1 | CgC4NY57 | Actin |
| **Myosin** | arpc1a | CgK1R488 | Actin-related protein 2/3 complex subunit 1A |
|  | vmhc | CgK1QRU8 | Myosin heavy chain, striated muscle |
|  | vmhcl | CgK1RSS3 | Myosin heavy chain, striated muscle |
|  | myo18ab | CgK1QE10 | Myosin-XVIIIa |
|  | tpm3 | CgK1QNV6 | Tropomyosin |
|  | zgc:66156 | CgK1QTC1 | Paramyosin |
|  | myh6 | CgK1R1B3 | Myosin heavy chain, striated muscle |
| **Rabs proteins** | rab33ba | CgK1QRK0 | Ras-related protein Rab-33B |
|  | rab1ba | CgK1R150 | Ras-related protein Rab-1A |
|  | rab7 | CgK1PZ08 | Ras-related protein Rab-7a |
|  | rab11a | CgK1QD28 | Ras-related protein Rab-11A |
|  | Rab14 | CgK1QC78 | Ras-related protein Rab-14 |
|  | rab21 | CgK1PDE3 | Ras-related protein Rab-21 |
|  | rab2 | CgK1QBM3 | Ras-related protein Rab-2 |
| **Chaperonin-containing T complex** | tcp1 | CgK1RAJ1 | T-complex protein 1 subunit alpha |
|  | hspd1 | CgK1Q5G6 | 60 kDa heat shock protein, mitochondrial |
|  | cct5 | CgK1RLC5 | T-complex protein 1 subunit epsilon |
